# Rapid, Selective, and Ultra-Sensitive Field Effect Transistor-Based Detection of *Escherichia coli*

**DOI:** 10.3390/ma17153648

**Published:** 2024-07-24

**Authors:** Liena Zaidan, Inna Novodchuk, Alexander H.Xu, Alexandru Nica, Saeed Takaloo, Christopher Lloyd, Reza Karimi, Joe Sanderson, Michal Bajcsy, Mustafa Yavuz

**Affiliations:** 1 Department of Electrical and Computer Engineering, University of Waterloo, Waterloo, ON N2L 3G1, Canada; 2 Waterloo Institute for Nanotechnology (WIN), University of Waterloo, Waterloo, ON N2L 3G1, Canada; 3 Biograph Sense Inc., Kitchener, ON N2R 1V1, Canada; 4 National Institute for Materials Science (NIMS), University of Tsukuba, Tsukuba 305-0044, Ibaraki, Japan; 5 Department of Mechanical and Mechatronics Engineering, University of Waterloo, Waterloo, ON N2L 3G1, Canada; 6 RETEGO Bio Labs LLC, West Bountiful, UT 84087, USA; 7 Department of Physics and Astronomy, University of Waterloo, Waterloo, ON N2L 3G1, Canada; 8 Institute for Quantum Computing, University of Waterloo, Waterloo, ON N2L 3G1, Canada

**Keywords:** graphene, FET, biosensor, *E. coli*

## Abstract

*Escherichia coli* (*E. coli*) was among the first organisms to have its complete genome published (Genome Sequence of *E. coli* 1997 Science). It is used as a model system in microbiology research. *E. coli* can cause life-threatening illnesses, particularly in children and the elderly. Possible contamination by the bacteria also results in product recalls, which, alongside the potential danger posed to individuals, can have significant financial consequences. We report the detection of live *Escherichia coli* (*E. coli*) in liquid samples using a biosensor based on a field-effect transistor (FET) biosensor with B/N co-coped reduced graphene oxide (rGO) gel (BN-rGO) as the transducer material. The FET was functionalized with antibodies to detect *E. coli* K12 O-antigens in phosphate-buffered saline (PBS). The biosensor detected the presence of planktonic *E. coli* bacterial cells within a mere 2 min. The biosensor exhibited a limit of detection (LOD) of 10 cells per sample, which can be extrapolated to a limit of detection at the level of a single cell per sample and a detection range of at least 10–10^8^ CFU/mL. The selectivity of the biosensor for *E. coli* was demonstrated using *Bacillus thuringiensis* (*B. thuringiensis*) as a sample contaminant. We also present a comparison of our functionalized BN-rGO FET biosensor with established detection methods of *E. coli* k12 bacteria, as well as with state-of-the-art detection mechanisms.

## 1. Introduction

*Escherichia coli* (*E. coli*) is a Gram-negative bacteria found in the human digestive tract. While most strains of *E. coli* are harmless, some strains can cause severe illness and be life-threatening, particularly in young children and those with compromised immune systems. *Escherichia coli* (*E. coli*) infection can be particularly life-threatening in young children and the elderly. Contaminated water, including that contaminated by *E. coli*, leads to the deaths of over 500,000 people annually. According to the Centers for Disease Control and Prevention (CDC), an estimated 265,000 *E. coli* infections occur annually in the United States [1]. The World Health Organization (WHO) estimates that up to 10% of patients infected with *E. coli* can develop a life-threatening disease such as hemolytic uremic syndrome (HUS) [2]. Among those who develop a life-threatening illness, the case-fatality rate can range from 3% to 5% [3]. Although survivors of HUS may recover from the acute illness, they can suffer from serious long-term health complications, including neurological complications such as seizures, stroke, and coma, which occur in approximately 25% of HUS patients [4,5].

*E. coli* infection in humans can be caused by consuming contaminated foods, such as raw vegetables, as evidenced by the *E. coli* lettuce outbreak in 2020 [6,7]. *E. coli* has also been found in isolated bodies of water, leading to waterborne transmission through contaminated drinking water and recreational waters [2,3]. Effective food screening before reaching consumers and quick, cost-effective testing of suspected water contaminations can significantly reduce the incidence of *E. coli* outbreaks, potentially saving many lives, particularly in developing nations [2,3].

Detecting *E. coli* before consumption is crucial for reducing *E. coli*-related illnesses and preventing severe health consequences, especially in vulnerable populations such as young children and the elderly. The early detection of even small concentrations of *E. coli* is vital for identifying fecal contamination and controlling infection sources to prevent the spread of this pathogen. Additionally, detecting *E. coli* is beneficial to the ongoing research in Anti-Microbial Resistance (AMR), and there is a need for a cheap and sensitive biosensor in the field [8].

A polymerase chain reaction (PCR) is widely used for detecting *E. coli* due to its sensitivity to *E. coli* DNA and rapidity. The limit of detection (LOD) for PCR can be as low as 40 CFU per reaction, with detection times typically ranging from a few hours to a day [9,10]. However, the presence of PCR inhibitors in a sample, such as humic acids, heavy metals, and complex polysaccharides, can affect the accuracy of the results [11,12,13,14]. Obtaining reliable results may thus require additional sample preparation steps, such as the co-purification or inactivation of inhibitors. In cases where the target *E. coli* concentration is low, performing a pre-enrichment step may also be necessary to improve the assay’s sensitivity. These additional steps can increase assay time and complexity [14].

An alternative method for detecting specific proteins or molecules is the enzyme-linked immunosorbent assay (ELISA). This method utilizes the particular interaction between antibodies and antigens, as well as the catalysis between the enzyme and substrate, to detect the target molecules [12,15]. ELISA is relatively simple to perform and can be used to detect multiple target molecules simultaneously. However, it may not be as sensitive as PCR or as capable of detecting specific gene sequences. The LOD for conventional ELISA (C-ELISA) for *E. coli* O157:H7 is typically between 10^5^ and 10^7^ CFU/mL, which is inadequate for detecting low concentrations of bacteria. However, novel ELISA methods, such as FNP-ELISA, have improved sensitivity with an LOD of 68 CFU/mL and a detection time of approximately 3 h [16].

Next-generation sequencing (NGS) is a high-throughput sequencing method that allows for the direct quantification of target bacteria by detecting specific genes [17]. The LOD for NGS in detecting anti-microbial resistance genes (ARGs) in *E. coli* can be achieved with a sequencing depth of ~15× coverage, requiring approximately 30 million reads for adequate sensitivity [18]. One of the significant advantages of NGS is its ability to rapidly generate large amounts of data at a relatively low cost per base. It can also detect the presence of multiple pathogens and resistance genes in a single sample by sequencing only a few kilobases of the genome. In addition, NGS kits often include software for strain typing. However, NGS has some limitations, including the inability to distinguish between live and dead bacteria and requiring highly trained personnel. The high cost and novelty of NGS methods can also be deterrents for some users.

One significant challenge in testing water for *E. coli* is that suspected contaminated samples may contain a wide variety of other contaminants besides the target bacteria. This can interfere with the selective and accurate detection of *E. coli*, making the testing process expensive, time-consuming, and difficult [19,20].

Recently, the use of functionalized field-effect transistors (FETs) for the detection of cancer markers [21,22], viruses [23], and bacteria [24] have shown that these types of biosensors can potentially be helpful as a platform for sensitive detection in healthcare applications. This diversity in applications underscores the specificity and sensitivity of FET biosensors across a range of health-related targets. For an insight into recent advancements, Table 1 below lists selected detection limits and specific target molecules that highlight the capabilities of these state-of-the-art FET-based biosensors. FET biosensors offer several advantages, including label-free detection, simplicity of operation, affordability, integration into portable applications, low power usage, high sensitivity, and easy fabrication, thus leading to high commercial viability [25]. Despite this, their limitations have been reported in the literature. Current FETs used to detect bacteria often have short detection ranges for *E. coli* K12 [26,27], require long detection times [27,28], or involve cumbersome processes to prepare linkers for binding and selectivity enhancement [29]. Therefore, further research is needed to improve the detection range, time, and sensitivity of the technology for *E. coli* detection.

In addition to experimental investigations in FET biosensors, simulation, modeling, and computational methodologies have also been significantly studied. This has enabled researchers to optimize their design and understand complex phenomena occurring in these devices. Ref. [30] presents a numerical model for FET biosensors that includes the effects of ionic charge screening and surface adsorption. The results showed that this model successfully assessed the impact of varying design parameters on the sensitivity of these devices. Ref. [31] reported a mathematical model of the graphene–electrolyte interface and graphene’s quantum capacitance for graphene electrolyte-gated FET. The simulation results were validated through experimental data to estimate device parameters: mobility, minimum carrier concentration, interface capacitance, contact resistance, and effective charged impurity concentration. In addition to these two articles, ref. [32,33] have also used computational techniques to improve the sensitivity and accuracy of biosensors by addressing the redistribution of ions and calculating non-faradic currents. Therefore, computational techniques have been shown to be effective in improving the performance of FET biosensors.

Among all designs of FET biosensors, 2D transition metal dichalcogenide (TMD)-based FETs and Graphene FET (G-FET) have been studied extensively due to their wide applications in the literature. Cao et al. presented an analytical current-voltage model for 2D TMD-based FETs [34]. Their model considered the physics of 2D TMD crystals and addressed significant effects, namely interface traps, mobility degradation, and inefficient S/D extension doping. Ref. [35] developed a compact model for predicting the electrical response of FET biosensors based on 2D semiconductors, e.g., MoS_2_. Their model considered the physics of electrostatics and charge density, site-binding modeling, and carrier transport inside an FET. A Verilog-A implementation, an alternative tool, was used to validate the results of simulations alongside experimental data from an MoS_2_ biosensor. The Verilog-A has also been used in [36] to develop a compact quasi-ballistic model for a dual-gated G-FET. Additionally, the challenge of baseline drift in G-FET biosensors and the stability of bioreceptors in liquid gate FET configurations has been addressed in [37], utilizing state-space models and a combination of computational and molecular biology techniques. Besides all these compact and analytical models, employing the Poisson–Boltzmann model for estimating the surface average of the potential in G-FETs has been addressed [38,39]. Ref. [32] reported modeling and simulations of probe-modified G-FETs for *E. coli* detection using COMSOL Multiphysics. They investigated the effects of *E. coli* motion and induced surface charge on graphene. They also found a relationship between source-drain current, graphene-bacteria distance, and bacterial concentration. Although TMD-based FETs and G-FETs have been widely studied, few studies have been devoted to modeling reduced graphene oxide-based FETs.

Herein, we utilize a FET biosensor with B/N co-coped reduced graphene oxide (rGO) gel (BN-rGO) [40] as the transducer material and functionalized with a bioreceptor derived from antibodies to demonstrate the detection of *E. coli* K12. Previously, a FET biosensor based on a functionalized BN-rGO transducer was used to detect low-concentration solutions of ‘pure’ biomarkers, such as a B-type natriuretic peptide [41], which can serve as a heart-attack marker, and COVID-19 nucleocapsid protein [42]. This work reports the first use of functionalized BN-rGO FET for the bio-detection of live-culture samples.

The device was functionalized with antibodies specific for the *E. coli* K-12 O-antigen and exhibited a low limit of detection (LOD) and an extensive detection range over eight orders of magnitude in a buffer solution. It also exhibited excellent selectivity against a live sample contaminant in the form of *Bacillus thuringiensis* and a detection time of less than two minutes.

**Table 1:** Selected Detection Limits and Target Molecules for Emerging State-of-the-Art FET-Based Biosensors.

Biomarker	LOD	Range	Detection Time	Reference
Carcinoembryonic antigen (CEA)	72 ag/mL	1 fg/mL–1 ng/mL	N/A	[43]
CA125 breast cancer biomarker	0.01 U/mL	0.01–1000 U/mL	~20 min	[44]
Prostate-specific antigen (PSA)	1 fg/mL	1 fg/mL–100 ng/mL	~2 min	[45]
SARS-CoV-2 S1 antigen	4.12 fg/mL	0.1 fg/mL–5.0 pg/mL	~2–3 min	[46]

## 2. Experimental Section

### 2.1. Materials

Hexagonal boron nitride (h-BN) ultrafine powder and a GO solution were purchased from Graphene Supermarket. A phosphate-buffered saline (PBS) solution, pH = 7.4, was purchased from Thermo Scientific. *Escherichia coli* and *Bacillus thuringiensis* were purchased from the American Type Culture Collection (ATCC 25404 and ATCC 33671, respectively). Both strains of bacteria were grown in a filter-sterilized Luria broth (LB) medium (1% tryptone, 0.5% yeast extract, and 1% NaCl) for 12 h at 37 °C in sterile cell culture shaker flasks at 200 rpm. Each bacterial culture was individually centrifuged at 3300× *g* for 15 min; the spent LB supernatant was decanted, and then each bacterial culture was resuspended and diluted in sterile phosphate-buffered saline (PBS) to the desired concentrations.

### 2.2. Fabrication of BN-rGO FETs

In our previous work [40], we demonstrated the fabrication of the BN-rGO gel FETs. Briefly, a solution containing GO and a solution containing h-BN quantum dots were mixed and irradiated using the pulses from a 1 kHz ultra-short femtosecond (fs) laser, with an average power of 1 W, focused by a 5cm lens to a spot area of 78.5 × 10^−5^ cm^2^, giving an intensity of 3.5 × 10^15^ W/cm^2^. The focus of the laser beam was held 2 mm below the solution’s surface for 50 min. The solution was stirred throughout the ablation process using a magnetic stirrer (Sigma-Aldrich, St.Louis, MO, USA), resulting in a BN-rGO gel. The BN-rGO gel was used as the channel material, with a channel width of 53 μm, between source and drain electrodes in a back-gated FET device with a pre-patterned Au/Ti source and drain electrodes on top of a SiO_2_/p-Si substrate.

### 2.3. Working Principle of BN-rGO FET Biosensor

In a typical FET biosensing system, biorecognition elements tailored explicitly for high specificity and binding affinity with the target are immobilized along the semiconductor pathways between the source and drain electrode. The system operates by applying and adjusting a bias potential via a gate electrode, which effectively modulates the conductance within the sensing channel. When the target analytes bind to the bioreceptors on the sensor surface, they induce a localized electric field. This field changes the charge distribution at the surface of the semiconductor channel. This conductance alteration, triggered by the interaction with target molecules, is then recorded and processed through a specialized electrical measurement system.

### 2.4. Antibody Functionalization and Device Passivation

In our previous investigation [42], a methodology akin to the one employed earlier was followed to affix anti-*E. coli* O-antigen antibodies onto the BN-rGO-functionalized channels. The choice of BN-rGO gel as a functionalizing agent was predicated on its unique characteristics, which include high mobility and abundant carboxyl groups. These features are instrumental for the covalent binding of antibodies through an amine–carboxyl reaction, a process thoroughly characterized [40]. The high mobility of the BN-rGO gel facilitates rapid electron transport, enhancing the sensor’s response time.

In contrast, the carboxyl group of the BN-rGO gel proved instrumental in facilitating the effective covalent binding of antibodies through an amine–carboxyl reaction, as explicated by [47]. The carboxyl groups enable strong covalent bonding with the antibodies’ amine groups, ensuring stable attachment. This chemical specificity allows antibodies to be precisely oriented and densely packed, maximizing the sensor’s ability to capture *E. coli* efficiently. The uniform distribution of these functional groups across the BN-rGO gel surface ensures an optimal environment for antibody immobilization, significantly enhancing the sensor’s performance by improving its sensitivity and selectivity in complex biological samples. Subsequently, 5 μL droplets of the antibody solution were meticulously pipetted onto the BN-rGO channel surface, allowing for a 48 h immobilization period at 4 °C. Rigorous rinsing with PBS, accomplished through successive 10 μL droplets of the buffer solution applied and removed five times, was performed to ensure robust immobilization. To mitigate non-specific binding, the devices underwent passivation via incubation in an ethanolamine solution, followed by thorough rinsing with PBS.

### 2.5. Immunodetection

Electrical measurements were performed with a probe station interfaced with a KEYSIGHT B2900A (4.2.2045.2760) Series software-controlled source measure unit. The reference measurement was performed using a pipette to drop the PBS buffer onto the channel of the FET biosensor. Solutions of different concentrations were made using a solution containing *E. coli* in 0.1× PBS buffer and serially diluting. In total, 2.5 μL of each *E. coli* solution was periodically dropped onto the BN-rGO channel to verify real-time measurement capabilities. Continuous monitoring of the device’s current was performed at fixed drain and back gate voltages of −0.05 V and −0.6 V, respectively. The Dirac point change was evaluated by dropping 2.5 μL of each *E. coli* diluted solution onto the channel of the biosensor, and a drain current vs. gate voltage plot was observed after 2 min. After each Dirac point measurement, the channel was rinsed with the PBS solution, and then the measurement was repeated with another solution of *E. coli* of higher concentration. The experiments were conducted using six different BN-rGO gel FET devices to ensure reproducibility and accuracy of the results.

For the selectivity experiments, the Dirac point measurement was performed using the same procedure described for *E. coli*, but instead of 0.1× PBS/*E. coli* solution, a solution of 0.1× PBS/*B. thuringiensis* was pipetted. The biosensor’s current was continuously monitored for constant gate and drain voltages of −1.5 V and −50 mV, respectively.

### 2.6. Device Configuration

**Figure 1:**
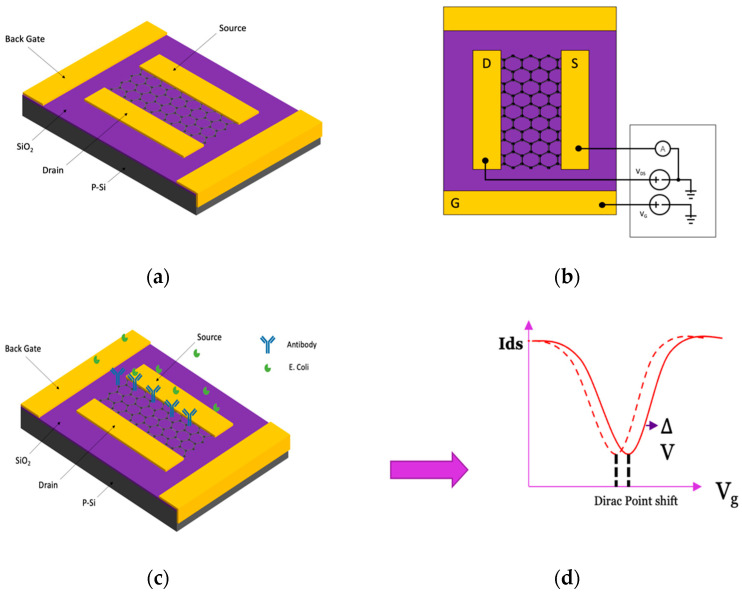
The schematics of the device configuration and biosensing mechanism of the BN-rGO gel *E. coli* biosensor. (**a**) The back-gated FET device configuration features the BN-rGO gel channel between the source and drain electrodes. (**b**) The circuit connections of the BN-rGO gel FET device. (**c**) The device configuration following *E. coli* antibody functionalization and passivation with ethanolamine. (**d**) The biosensing response is determined by the shift in the Dirac voltage after introducing the *E. coli*-containing solution.

This study’s employed FET device configuration mirrors our prior publications [42], a p-doped silicon wafer with a SiO_2_ dielectric layer as the substrate, source, and drain electrodes. Additionally, it incorporates a BN-rGO gel channel, as depicted in Figure 1a. To enable the specific detection of *E. coli* bacteria within a sample, the device undergoes functionalization with *E. coli* antibodies. Subsequently, the device is passivated with ethanolamine to eliminate any undesirable charge-related effects originating from the sample, as demonstrated in Figure 1b [48].

## 3. Results and Discussion

### 3.1. Sensing Performance

**Figure 2:**
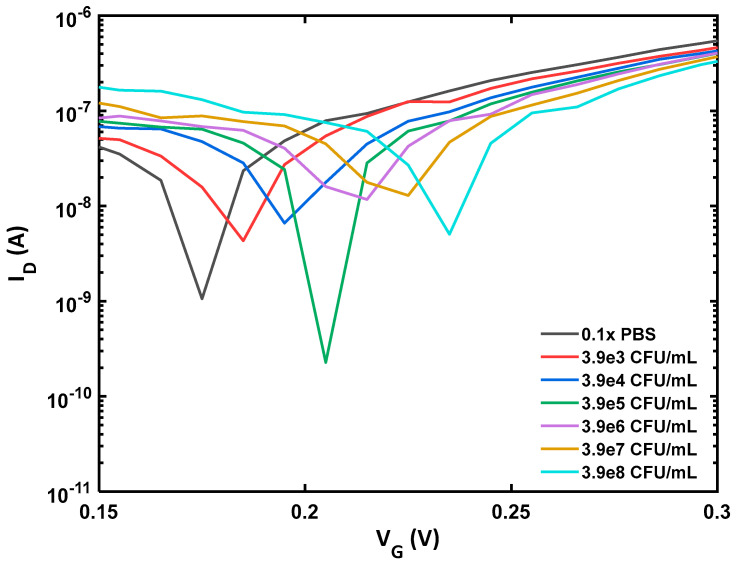
*E. coli* biosensing performance of antibody-functionalized BN-rGO gel FET. Shift in Dirac and increase in ON-current correspond to increasing concentrations of *E. coli* in solution.

The *E. coli* cells were diluted in a PBS buffer solution. The drain current for BN-rGO gel FET, functionalized with antibodies and passivated, was graphed against gate voltage while maintaining a constant drain voltage of −50 mV. In this measurement, we used a sterile buffer solution to represent a baseline condition in which no *E. coli* cells were present in the sample. Various concentrations of *E. coli* diluted in a buffer were deposited onto the channel for sensing experiments, and the drain current vs. gate voltage was monitored. *E. coli* has a net negative charge around its membrane [29]; their binding to the anti-*E. coli* will result in an augmentation of negative charge within the BN-rGO gel. This will induce hole carriers in the BN-rGO channel and increase the Dirac voltage, as illustrated in Figure 1d. The performance of the BN-rGO gel FET biosensor was evaluated by monitoring its response to varying concentrations of *E. coli* K-12. As demonstrated in Figure 2 (Figures S1–S6), a slight change in concentration results in a difference in the ON-current and a shift in the Dirac voltage, underscoring the remarkable sensitivity of the BN-rGO gel FET biosensor. This trend is caused by *E. coli* cells accumulating in the sample solution; the negative bound bacterial cells function as gate electrolytes, generating a negative potential. Thus, more hole carriers are induced, leading to a gradual increase in the ON-current.

The increased off-current with higher concentrations of *E. coli* can generally be attributed to the enhanced negative potential near the channel induced by the binding of negatively charged *E. coli* cells. This interaction effectively lowers the energy barrier for hole conduction even when the device is supposed to be in the ‘off’ state, resulting in an increased off-current. However, there were exceptions at two specific concentrations where this trend did not follow. This anomaly could be due to factors such as an uneven distribution of *E. coli* cells across the sensor surface, aggregation of bacteria leading to reduced effective surface interaction, or variations in the bioactivity of the *E. coli* cells at different concentrations.

### 3.2. Sensitivity

The BN-rGO gel FET biosensor’s ability to detect different concentrations of *E. coli* is crucial for its effective performance. Sensitivity in this context refers to the ability to detect very low concentrations and how the device responds to varying levels of the target analyte, which can be observed through changes in the Dirac voltage and fluctuations in the ON-current [41,47].

**Figure 3:**
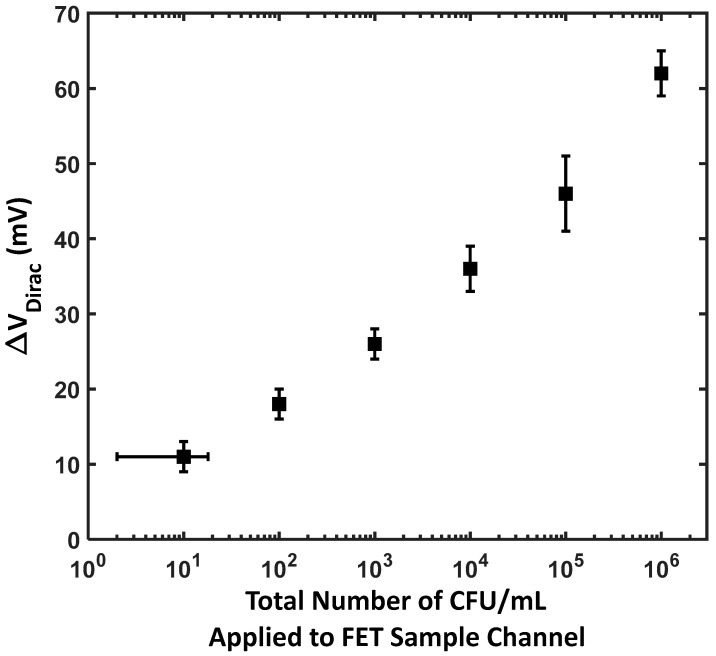
The shift in the Dirac voltage in response to different CFU/mL *E. coli* concentrations.

As *E. coli* bacteria bind to the antibody-functionalized surface of the BN-rGO gel, the sensor experiences an increase in negative charge accumulation. This accumulation influences the charge carrier density within the channel, leading to a shift in the Dirac voltage. Specifically, the relationship between the *E. coli* concentration and the Dirac voltage shift follows a logarithmic trend, represented by the equation ${∆V}_{Dirac}$ = 3.803*ln (*E. coli* concentration) +2.716 (Figure 3). This logarithmic response indicates that the sensor’s sensitivity allows it to detect a wide range of *E. coli* concentrations with high precision. The OFF-current is observed to increase with higher *E. coli* concentrations. This can be attributed to multiple factors, including the increased charge carrier density and surface states and trap charges due to the increased bound bacterial cells on the antibody-functionalized surface. Additionally, the electric field within the FET is modulated due to the electrostatic interactions of the negatively charged bacterial cells and the channel.

The BN-rGO gel FET biosensor demonstrates a low LOD, capable of identifying concentrations as low as one cell per sample. This high sensitivity is achieved due to the efficient charge transfer properties, the BN-rGO gel’s high carrier mobility, and the specific binding interactions between the *E. coli* antibodies and the bacterial cells.

### 3.3. Selectivity and Specificity

Ensuring the biosensor’s selectivity and specificity is crucial for its reliability and applicability. The biosensor’s selectivity relies on the receptor’s ability to exclusively bind to the target biomolecules, minimizing interference from other coexisting molecules in the biological sample [49].

In the context of clinical diagnoses, actual biological samples are inherently complex, containing diverse biomolecules. The biosensor’s specificity establishes whether the recognition interaction will be exclusively between the analyte and the bioreceptor [50].

**Figure 4:**
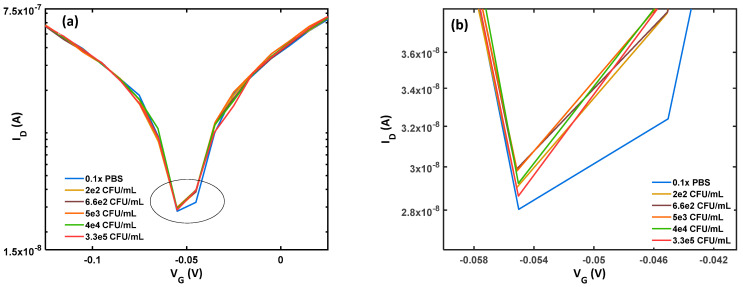
Specificity and selectivity response of BN-rGO gel FET *E. coli* biosensor towards the blank sample and *B. thuringiensis*. (**a**) Gate voltage in the range of −0.15 to 0.025 V, (**b**) gate voltage in the range of −0.06 to −0.04 V.

Different concentrations of *B. thuringiensis* were added to the functionalized BN-rGO gel channel to examine the BN-rGO gel FET biosensor selectivity and specificity. The Dirac shift in response to the different concentrations determines the sensor’s selectivity and specificity, as shown in Figure 4. Notably, the selectivity assessment of the device involves comparing the Dirac voltage response to *B. thuringiensis* with the response to *E. coli* in the LOD concentration (10 CFU/mL). As seen in Figure 4, the Dirac voltage for increasing concentrations of *B. thuringiensis* was approximately 4 mV, and the biosensor’s response was weak with a 2.5 times lower response, similar to the biosensor’s response to a blank sample with only 0.1× PBS buffer. This outcome demonstrates the BN-rGO gel FET biosensor’s excellent selectivity and specificity for the intended target *E. coli*.

### 3.4. Performance Compared to the State of the Art

In addition to selectivity and specificity, the parameters of the LOD and detection range play a critical role in assessing the biosensor’s performance [42]. A broad detection range allows the biosensor to detect *E. coli* over a wide concentration range, making it versatile and applicable to various sample types. This is especially valuable in clinical diagnostics, environmental monitoring, and food safety, where *E. coli* concentrations vary significantly. In addition, wide detection ranges provide a more comprehensive view of the sample’s *E. coli* concentration. It allows for accurate quantification across different concentration levels, ensuring reliable results regardless of whether the concentration is high or low.

The LOD for the BN-rGO gel FET biosensor is discernible in Figure 3. It indicates a detection threshold of one cell per sample, accompanied by a significant Dirac voltage shift of 5 mV. This demonstrates that the sensor can detect extremely low concentrations of *E. coli*, as even slight increases in bacterial concentration cause noticeable shifts in the Dirac voltage. The shift in the Dirac voltage was extracted for different concentrations of *E. coli* in the buffer solution, and the results are summarized in Figure 3. The binding of analyte molecules to the FET surface follows the Langmuir adsorption isotherm, which relates the fractional surface coverage to the analyte concentration. On a logarithmic scale, the Langmuir isotherm exhibits a linear region over a certain concentration range [51].

The BN-rGO FET biosensor stretches over a wide range of concentrations from 10 to 10^8^. It is worth highlighting that when presented on a logarithmic scale, the connection between the alteration in the Dirac voltage and concentration remained consistently linear and did not exhibit saturation even at the tested concentrations, indicating that the detection range may be longer than originally tested. The results were fitted ($R^{2}$ = 0.98) with the following relation: ${∆V}_{Dirac}$ = 3.803*ln (*E. coli* concentration) +2.716.

In comparison to prior studies (Table 2), it is evident that none have presented a comprehensive biosensor encompassing a balanced combination of key performance parameters, including the LOD, detection range sensitivity, and detection time. For instance, ref. [26]’s work yielded a small LOD and rapid detection time yet suffered from a limited detection range. Furthermore, ref. [28] reported a somewhat low LOD and a broad detection range but was plagued by extended detection times. Ref. [27] developed a portable microfluidic biosensor for the specific detection of *E. coli* (O157:H7), utilizing a finger-actuated mechanism. While this microfluidic biosensor facilitated a “sample-in and answer-out” assay for *E. coli* (O157:H7) and eliminated the need for external pumps and specialized personnel, it exhibited a relatively long detection time and a limited dynamic range spanning six orders of magnitude. These characteristics may influence its practicality for real-time detection in specific applications.

Conversely, ref. [29] achieved a low LOD and a somewhat broader detection range; however, their biosensor’s preparation process was notably complex, exceeding the complexity of our biosensor’s fabrication. Their study reported the development of an improved graphene-based FET modified with heat-denatured casein (GFET) for the sensitive detection of *E. coli* O157:H7 in natural water samples. The heat-denatured casein, coated on the graphene surface, served a dual purpose as a probe linker, facilitating binding with the specific antibody of *E. coli* O157:H7, and as a blocker to prevent interference signals stemming from non-specific adsorption between the graphene surface and impurities present in natural water samples.

Consequently, our biosensor demonstrates superiority when considering the overall sensing performance despite not achieving the absolute minimum LOD observed in certain studies. This underscores the holistic advantage of our biosensor in addressing a broader range of practical applications. In the present study, the determined LOD was influenced by Debye screening effects. Forthcoming research endeavors must be directed toward addressing and mitigating this particular challenge. Furthermore, the focus of future investigations should extend to the practical application of our biosensor in real-world samples, such as water and food matrices, for the robust and accurate detection of *E. coli*.

**Table 2:** Biosensing performances of other state-of-the-art *E. coli* (O157:H7) biosensors.

Method	LOD (CFU/mL)	Range (CFU/mL)	Sample	Detection Time	Reference
Carbon dots–Fe_3_O_4_ nanomaterial	6.88	10–10^8^	Milk and water	~35–40 min	[28]
Portable microfluidic biosensor with finger actuation	10	10^2^–10^8^	Buffer	~2.5 h	[27]
rGO-based field-effect transistor	1.4	1.4–1.4^7^	Buffer	~46 s	[26]
Graphene-based field-effect transistor	1	1–10^7^	River water	<3 min	[29]
BN-GO gel-functionalized field-effect transistor	**10**	**10–10^8^**	**Buffer**	**<2 min**	**This work**

## 4. Conclusions and Future Outlook

In summary, we have successfully developed a graphene-based field-effect transistor (FET) biosensor capable of detecting live *E. coli* in buffer solutions. The underlying detection principle relies on a graphene-based semiconductor formed by a Boron Nitride-reduced Graphene Oxide (BN-rGO) gel, covalently functionalized with antibodies specific to *E. coli*. Our biosensor device exhibited an impressive limit of detection (LOD) as low as one cell within a concise time frame of 2 min. Moreover, the biosensor demonstrated an exceptional detection range of 8 orders of magnitude from 10 to 10^8^ CFU/mL. Notably, the BN-rGO gel FET biosensor exhibited excellent specificity and selectivity and minimal interference from other substances.

These findings underscore the significant potential of BN-rGO gel FET biosensors for the rapid label-free detection of *E. coli*. Future investigations should aim to extend the applicability of this biosensor to more complex sample matrices, such as water or food. This will test the robustness and versatility of the biosensor under varied and potentially challenging conditions. Additionally, efforts to enhance the sensitivity and detection range could involve exploring alternative functionalization materials or improving the integration of the BN-rGO gel with other biorecognition elements. Furthermore, future studies should focus on analyzing potential interferences by testing the biosensor against various families of bacteria with similar surface charges to assess their effects on the biosensor’s selectivity and specificity. In addition, it is recommended that further studies be conducted in order to simulate the voltage–current model of the BN-rGO gel FET biosensors and validate theoretical results using the results of this study.

Developing portable and user-friendly versions of the biosensor for on-site testing is another crucial step that would enable real-time, on-the-ground monitoring of bacterial contamination, which is particularly valuable in remote or resource-limited settings.

This study serves as a proof-of-concept demonstration of the effective functionalization of BN-rGO gel with antibodies for bacteria detection, highlighting the broader potential of BN-rGO gel FETs for highly sensitive biosensing applications. Exploring these possibilities promises to further the field of microbial detection and enhance public health safety measures.

## Data Availability

The original contributions presented in the study are included in the article/Supplementary Material, further inquiries can be directed to the corresponding author/s.

## Supplementary Materials

The following supporting information can be downloaded at: https://www.mdpi.com/article/10.3390/ma17153648/s1, Figure S1: The shift in Dirac voltage as a response to 3.9 × 10^3^ CFU/mL *E. coli*; Figure S2: The shift in Dirac voltage as a response to 3.9 × 10^4^ CFU/mL *E. coli*; Figure S3: The shift in Dirac voltage as a response to 3.9 × 10^5^ CFU/mL *E. coli*; Figure S4: The shift in Dirac voltage as a response to 3.9 × 10^6^ CFU/mL *E. coli*; Figure S5: The shift in Dirac voltage as a response to 3.9 × 10^7^ CFU/mL *E. coli*; Figure S6: The shift in Dirac voltage as a response to 3.9 × 10^8^ CFU/mL *E. coli*.
