# Supplementary material for: Rapid, Selective, and Ultra-Sensitive Field Effect Transistor-Based Detection of Escherichia coli
Source: Materials (Basel). 2024 Jul 24;17(15):3648. doi: 10.3390/ma17153648 (PMC11313016; doi:10.3390/ma17153648)
Supplement: Supplementary file 1 [file materials-17-03648-s001.zip › materials-3092535-supplementary.pdf]

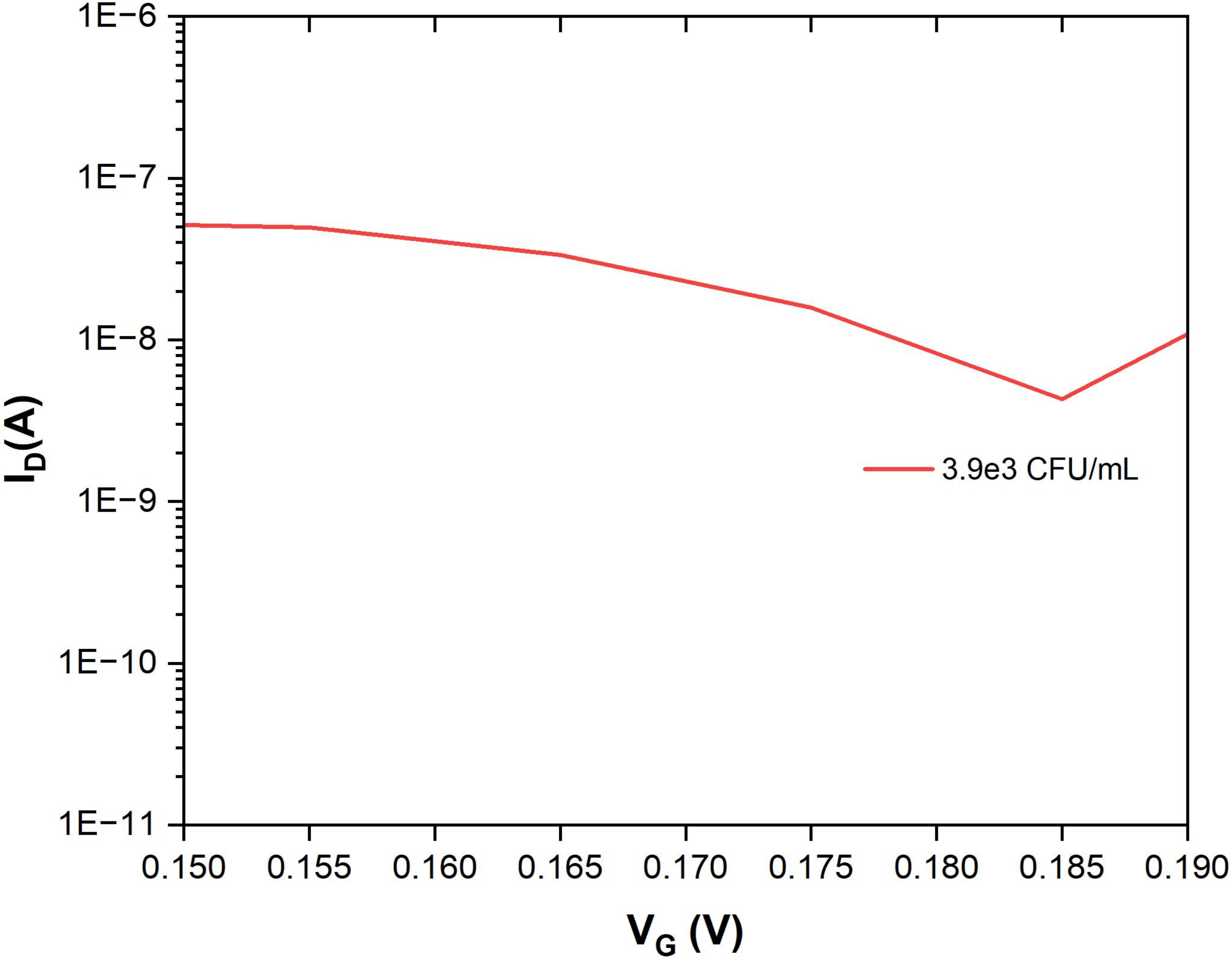

Figure S1. The shift in Dirac voltage as a response to 3.9e3 CFU / mL *E.coli.*

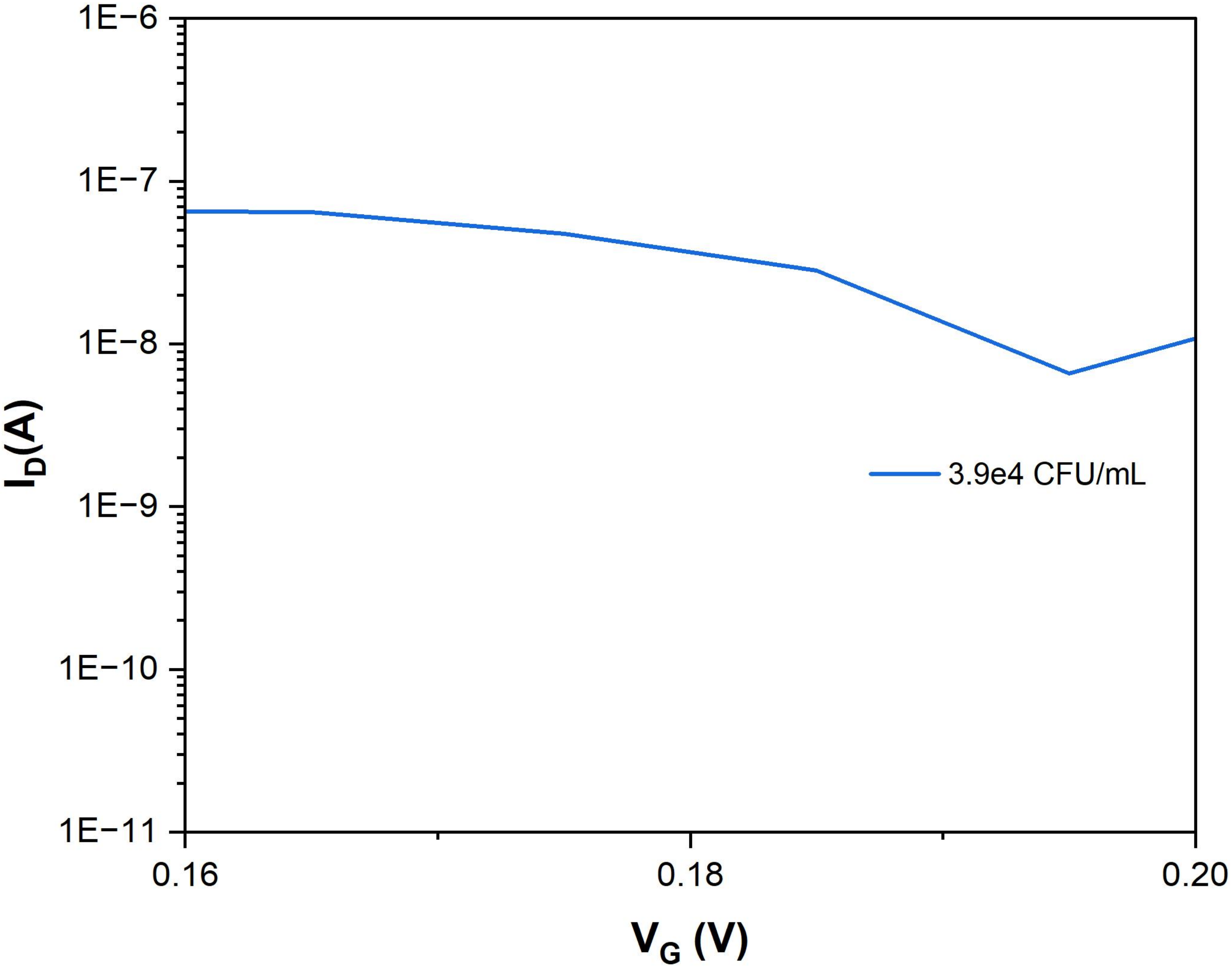

Figure S2. The shift in Dirac voltage as a response to 3.9e4 CFU / mL *E.coli*.

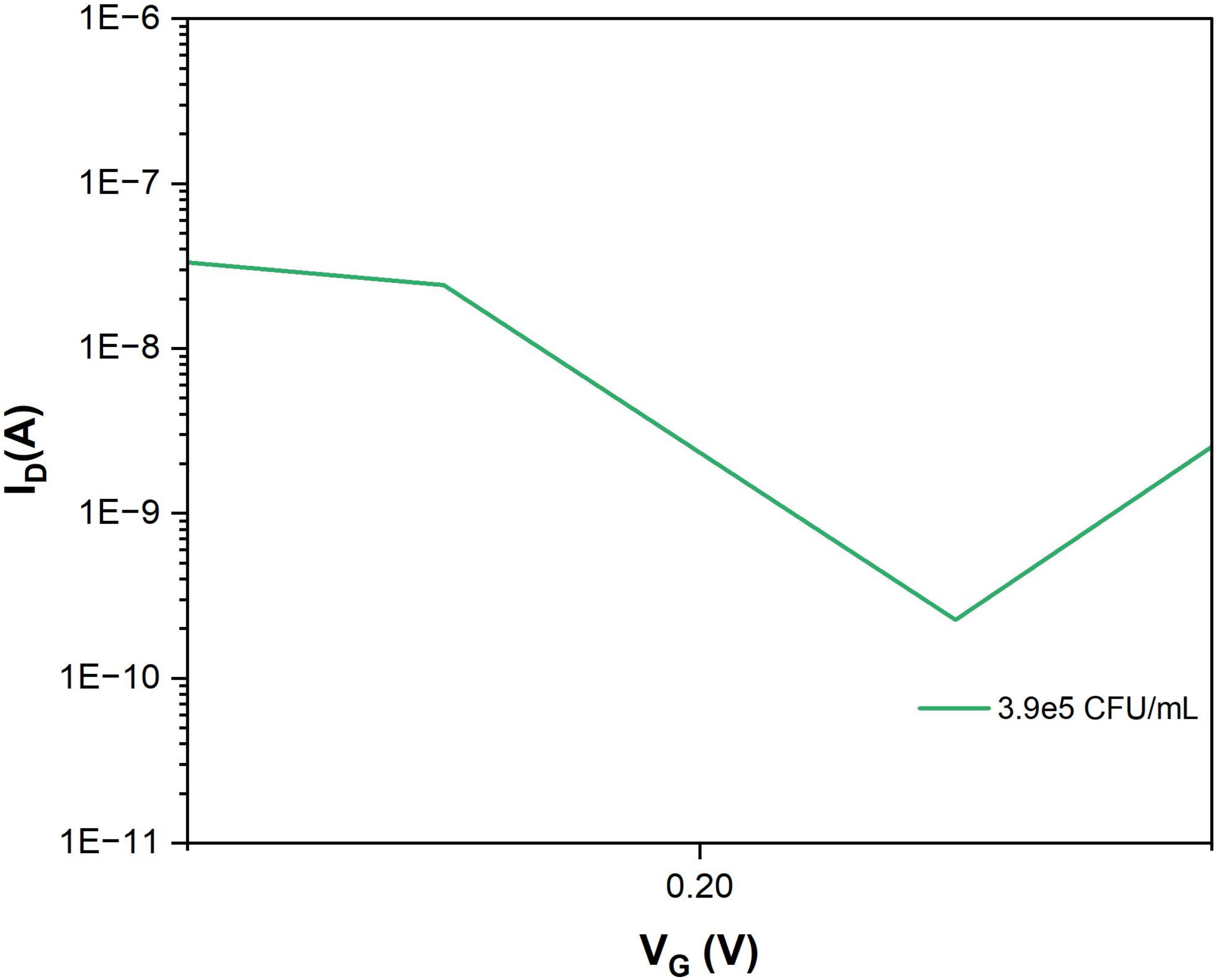

Figure S3. The shift in Dirac voltgae as a response to 3.9e5 CFU / mL *E.coli*.

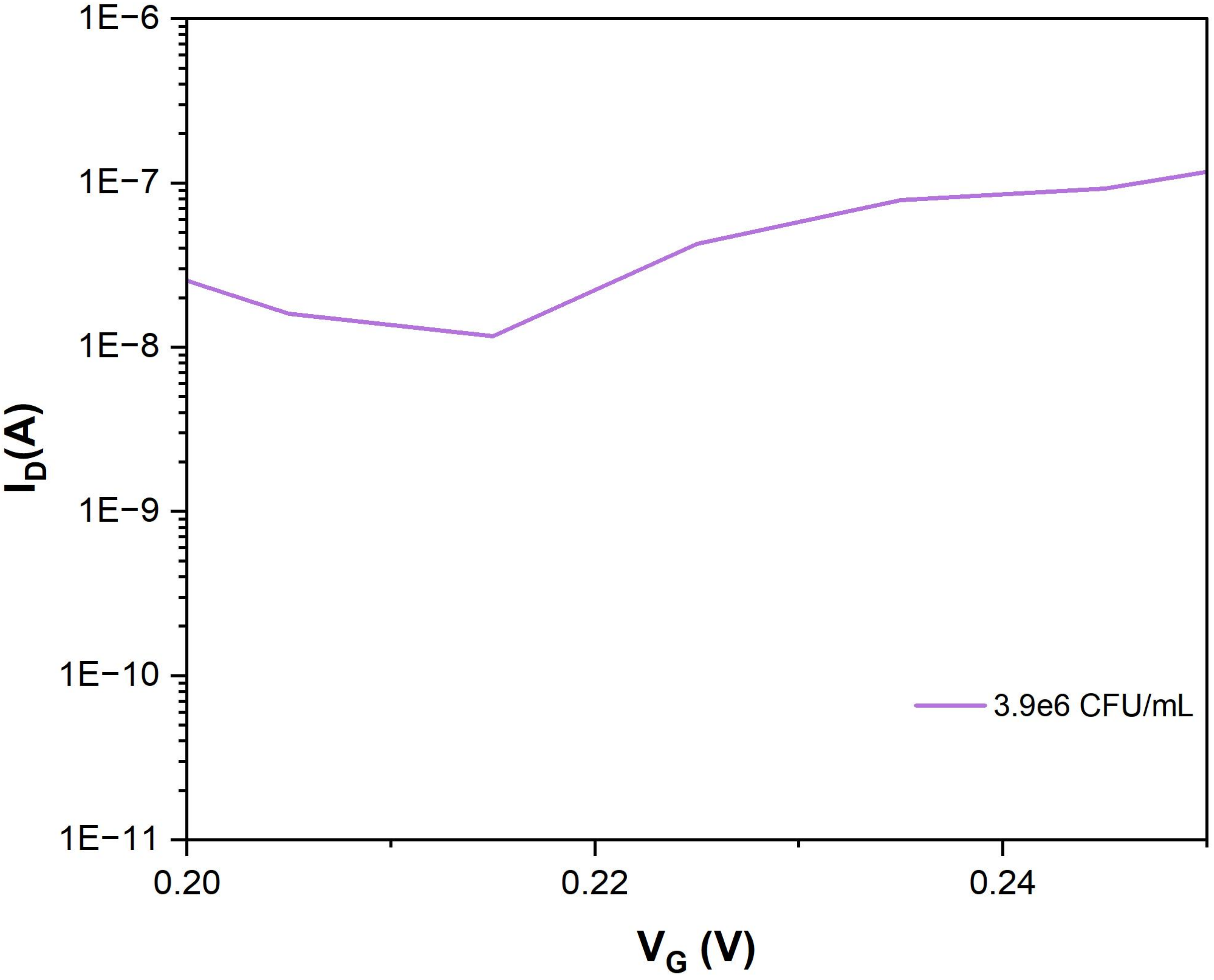

Figure S4. The shift in Dirac voltage as a response to 3.9e6 CFU / mL *E.coli*.

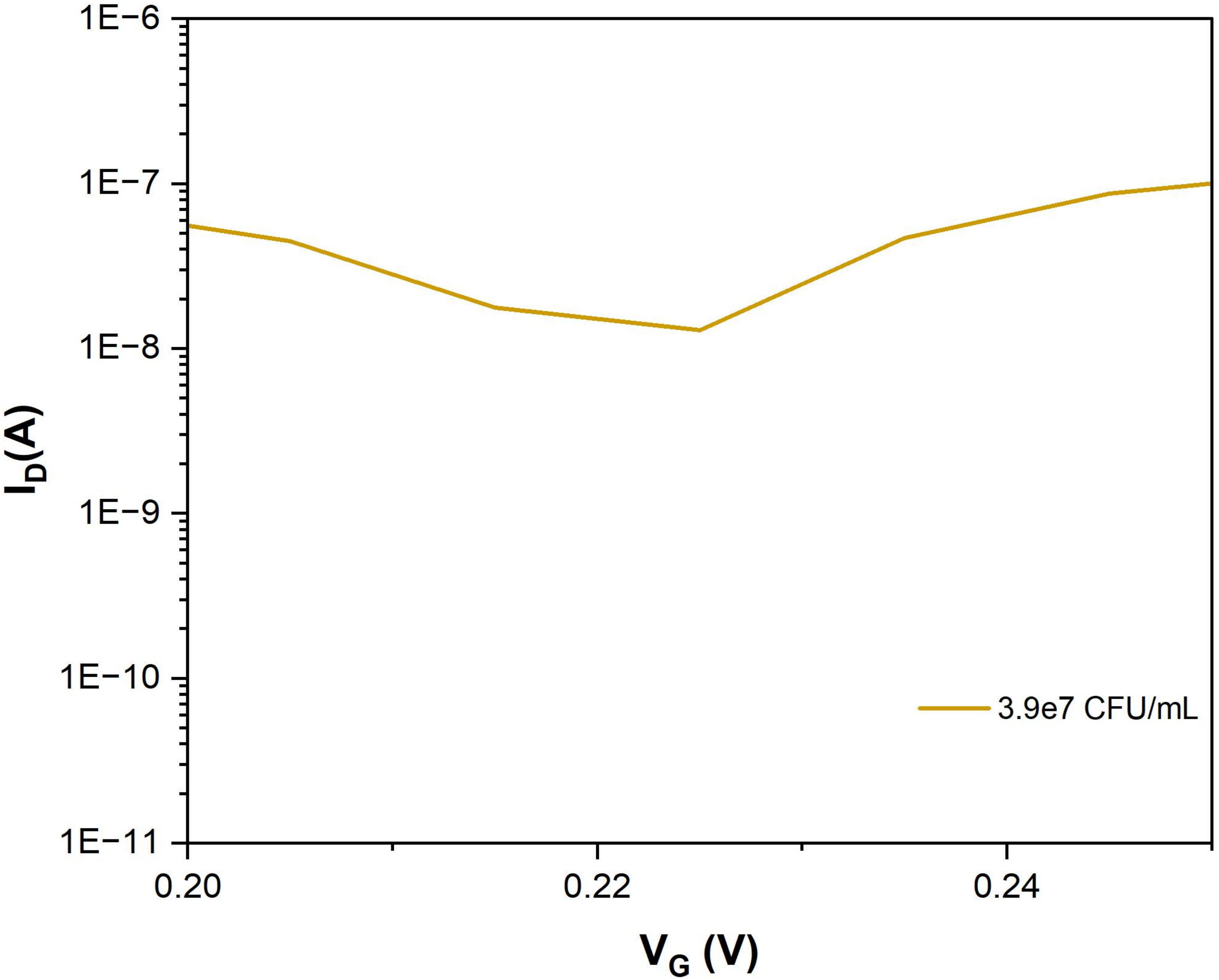

Figure S5. The shift in Dirac as a response to 3.9e7 CFU / mL *E.coli*.

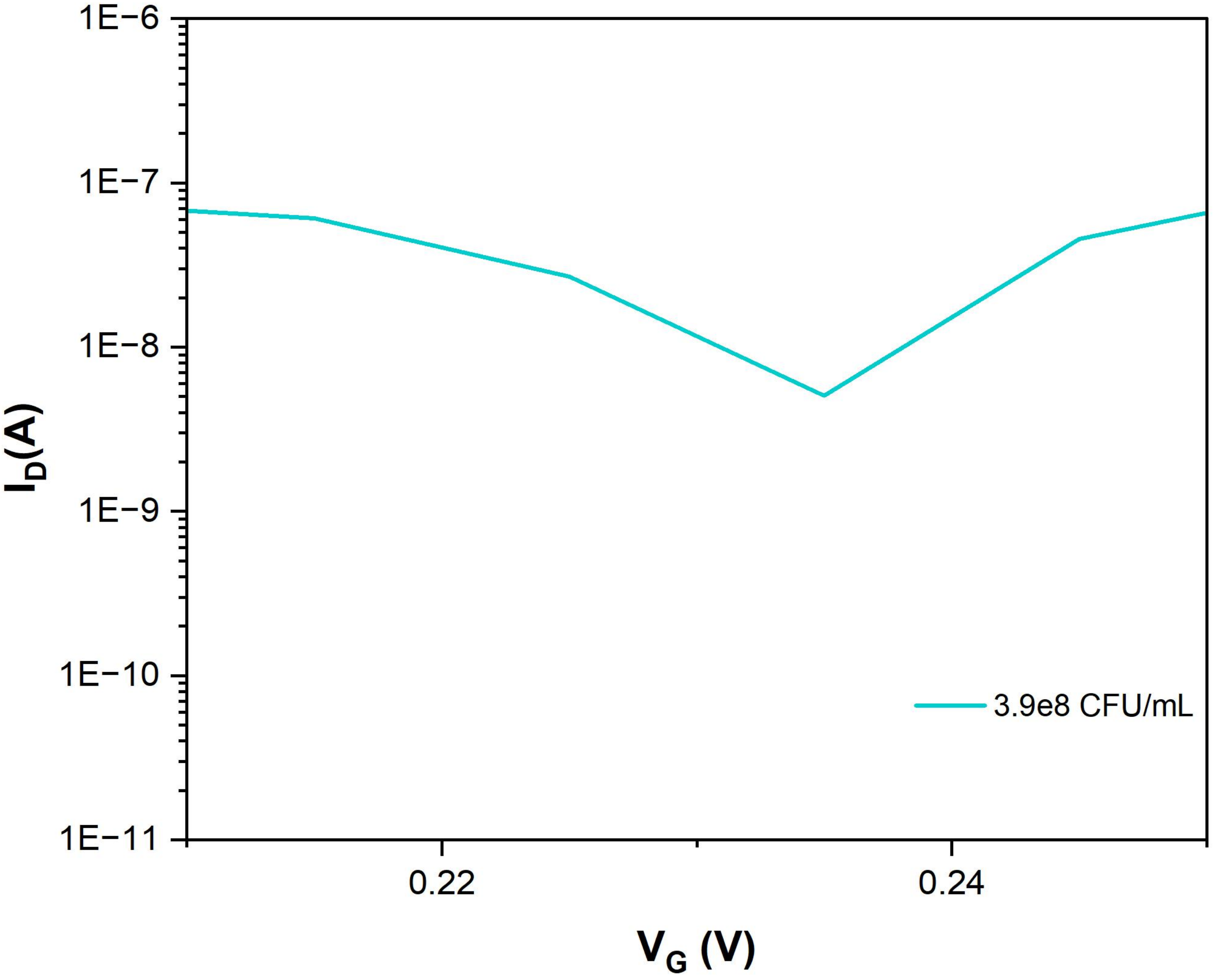

Figure S6. The shift in Dirac voltage as a response to 3.9e8 CFU / mL *E.coli*.
